# miR-144 attenuates the host response to influenza virus by targeting the TRAF6-IRF7 signaling axis

**DOI:** 10.1371/journal.ppat.1006305

**Published:** 2017-04-05

**Authors:** Carrie M. Rosenberger, Rebecca L. Podyminogin, Alan H. Diercks, Piper M. Treuting, Jacques J. Peschon, David Rodriguez, Madhumati Gundapuneni, Mitchell J. Weiss, Alan Aderem

**Affiliations:** 1 Center for Infectious Disease Research, Seattle, WA United States of America; 2 Department of Comparative Medicine, School of Medicine, University of Washington, Seattle, WA, United States of America; 3 Institute for Systems Biology, Seattle, WA, United States of America; 4 Hematology, St. Jude Children's Research Hospital, Memphis, TN United States of America; University of Pennsylvania, UNITED STATES

## Abstract

Antiviral responses must rapidly defend against infection while minimizing inflammatory damage, but the mechanisms that regulate the magnitude of response within an infected cell are not well understood. miRNAs are small non-coding RNAs that suppress protein levels by binding target sequences on their cognate mRNA. Here, we identify miR-144 as a negative regulator of the host antiviral response. Ectopic expression of miR-144 resulted in increased replication of three RNA viruses in primary mouse lung epithelial cells: influenza virus, EMCV, and VSV. We identified the transcriptional network regulated by miR-144 and demonstrate that miR-144 post-transcriptionally suppresses TRAF6 levels. *In vivo* ablation of miR-144 reduced influenza virus replication in the lung and disease severity. These data suggest that miR-144 reduces the antiviral response by attenuating the TRAF6-IRF7 pathway to alter the cellular antiviral transcriptional landscape.

## Author summary

Antiviral responses must be regulated to rapidly defend against infection while minimizing inflammatory damage. However, the mechanisms for establishing the magnitude of response within an infected cell are incompletely understood. miRNAs are small non-coding RNAs that negatively regulate protein levels by binding complementary sequences on their target mRNA. In this study, we show that microRNA-144 impairs the ability of host cells to control the replication of three viruses: influenza virus, EMCV, and VSV. We identify a mechanism underlying the effect of this microRNA on antiviral responses. microRNA-144 suppresses TRAF6 levels and impairs the gene expression program regulated by the transcription factor IRF7. The resulting dysregulated expression of antiviral genes correlates with enhanced viral replication. Our findings in isolated lung epithelial cells were consistent with the effects observed in influenza virus-infected mice lacking miR-144. Together, these data support a role for miRNAs in tuning transcriptional programs during early responses to viral infection.

## Introduction

Viruses co-opt host cellular processes in order to replicate, and pathogenicity often correlates with growth rate. The best-characterized antiviral program is regulated by type I interferons, which restricts multiple aspects of the viral life cycle (reviewed in [1–3]). An antiviral response program expressed at a level appropriate to the pathogenicity of the virus can effectively control infection. In contrast, an inadequate response will fail to restrain viral replication while an exaggerated inflammatory response can itself cause damage to the host. Ensuring rapid yet measured antiviral responses at mucosal surfaces, which require a threshold that permits containment of pathogenic insults yet tolerates benign foreign stimuli, is particularly important. In the case of influenza infection, progeny virions can be produced within 6 hours, necessitating a rapid response to quell the infection without triggering excessive inflammation that would compromise airway function.

MicroRNAs (miRNAs) are post-transcriptional regulators that are excellent candidates for finely tuning immune responses. These small (20–25 nucleotide) non-coding RNAs bind to target mRNAs by base-pairing to effect mRNA degradation or translational repression [4]. While the effect of miRNAs on individual target mRNA levels can often be modest, miRNAs are predicted to affect multiple targets in a biological pathway. This functional coherence in target genes can result in larger effects of a miRNA on biological processes than suggested by studies of individual miRNA-target interactions [5]. miRNA cooperativity in regulating levels of multiple targets in a pathway can also extend to cooperative interactions between multiple miRNAs in binding the same target, which can result in additive effects on biological processes [5, 6]. The ability of miRNAs to shape host-virus interactions is a recently emerging concept (reviewed in [7],[8, 9]). For example, miR-122 directly interacts with the hepatitis C (HCV) viral genome to stimulate translation and accelerate growth [10, 11]. miRNAs that can target host mRNAs to modulate antiviral responses following influenza infection have recently been identified [9]. However, the mechanisms by which host miRNAs shape antiviral resistance by controlling innate immune signaling pathways are poorly understood. Here, we demonstrate that miR-144 attenuates a module of antiviral interferon-induced genes controlled by TRAF6 and IRF7.

## Materials and methods

### Mice

C57BL/6 mice (Jackson Laboratories), IRF7^null^ mice (C57BL/6 background, provided by T. Taniguchi, University of Tokyo, Tokyo, Japan), and miR-144/miR-451^null^ mice were housed in a specific pathogen-free barrier facility. miR-144/miR-451^null^ mice [12] were backcrossed 5 times onto the C57BL/6 background (verified to be >95% C57BL/6), and wild type littermates used for infection experiments.

### Ethics statement

This study was carried out in strict accordance with the recommendations in the Guide for the Care and Use of Laboratory Animals of the National Institutes of Health and all efforts were made to minimize suffering and mice were euthanized by CO_2_ inhalation. All animal work was reviewed and approved by the Institutional Animal Care and Use Committee at the Center for Infectious Disease Research (protocol #AA-10) following guidelines established by the Institute of Laboratory Animal Resources and approved by the Governing Board of the U.S. National Research Council.

### Flow cytometry of uninfected lungs

Perfused lungs from C57BL/6 mice were digested using a dispase-agarose protocol [13] and cells isolated using CD45^+^ microbeads and autoMACS separation or sorting using a FACSAria. Live (7AAD^-^) cells were sorted from lungs as follows: CD45^+^Sca-1± (hematopoietic and bronchoalveolar stem cells), CD45^-^Sca1^-^T1a^+^ (type I epithelial), CD45^-^Sca1^-^T1a^-^ FITC (autofluorescence)^+^ (type II epithelial), CD45^-^Sca1^-^T1a^-^FITC^-^ (contains club cells). The sorted type II epithelial cells were predominantly pro-SPC^+^ (Millipore) and the club cells were predominantly Mucin^+^ (NeoMarkers), which identifies goblet cells in addition to club cells; by intracellular staining and immunofluorescence microscopy. To specifically exclude RBCs, enzymatically dissociated perfused lungs were treated with ACK lysis buffer, stained for Ter119, and Ter119^-^CD45^+^ (hematopoietic) cells and Ter119^-^CD45^-^T1alpha^+^ (type I lung epithelial) cells isolated using a FACSAria. Antibodies were obtained from e-Biosciences (T1alpha, Ter119) or BD (CD45, Sca1).

### miRNA profiling

miRNA profiling was performed on lungs infected with PR8 influenza virus for 72 h using 384-well microfluidic miRNA arrays (ThermoFisher) and miRNA expression measured relative to sno202 or U6. RNA was isolated using Trizol (Invitrogen) and cDNA synthesized from DNase-treated RNA using random primers or miR-specific primers and analyzed by qRT-PCR using gene- or miRNA-specific Applied Biossytems TaqMan primers and probes (ThermoFisher). Expression was normalized to EF-1 for mRNA and sno202 or U6 for miRNA.

### Viruses

Influenza A/Puerto Rico/8/34 was provided by P. Thomas (St. Jude’s, Memphis, TN), and VSV and EMCV obtained from ATCC.

### *In vivo* infection and quantification of viral load

Wild-type mice were anesthetized using ketamine/xylazine and challenged with 10^5^ PFU of PR8 influenza virus (for 12 h and 24 h time points) or 700 PFU (for 3–12 day time points) in 30 μL intranasally, divided between 2 nostrils. Weight-loss was measured daily as a correlate of morbidity. Lungs were lavaged using 1 mL of Hank’s buffered saline, the left lung lobe reserved for histopathological analysis, and the right lung lobes mechanically homogenized in 1 mLTrizol and RNA isolated for quantification of viral M gene by qRT-PCR as described for *in vitro* infections. 100 uL of cell-free lavage fluid was mixed with 1 mL Trizol for quantification of viral M gene. IRF7^null^ mice were challenged with 10^5^ PFU PR8 influenza virus for 24 h prior to lung lavage for viral load determination and lung RNA isolation for gene expression analysis as for WT animals.

### Flow cytometry on infected mice

Formalin-fixed cells were permeabilized using 0.2% saponin, stained with anti-influenza NP (ViroStat or Argene) antisera followed by fluorescently conjugated secondary antibodies, and fluorescence measured using a FACSCalibur. Infected lungs were enzymatically dissociated using Liberase Blendzyme III (Roche) and analyzed using a FACSCalibur. All cell lineage-specific antibodies used in S3 Fig were from BD except for PDCA-1 (Miltenyi).

### Histology

Five-micrometer sections from neutral buffered formalin-fixed left lung lobes were stained with hematoxylin and eosin and examined by a board-certified veterinary pathologist who was blinded to genotype and infection time point. Lung architecture was assessed to be normal in uninfected miR-144/miR-451^null^ mice. Histopathological severity was assessed on a four-point scale and the extent of the section affected in the most severe manner was scored on a 4 point scale. Histology scores were calculated by multiplying each severity metric by the extent of section affected in the most severe manner, with a maximum severity score of 16 for each histopathological feature. Pneumonia = interstitial pneumonia/alveolitis x extent of the most severe changes; Bronchial necrosis = necrotizing bronchiolitis x extent of the most severe changes; Acute inflammation = necrosuppurative bronchiolitis + perivascular neutrophils; Chronic inflammation = bronchial hyperplasia + alveolar hyperplasia + perivascular mononuclear cells + lymphoid aggregates.

### Cell lines

TC-1 C57BL/6 mouse lung epithelial cells, 293T, and Vero cells (ATCC) were used where indicated. LET1 cells were generated by murine stem cell virus (MSCV)-SV40 large T antigen transduction of primary mouse lung epithelial type I cells trans-differentiated from type II epithelial cells and used after a minimal number of passages (BEI Resources) [14].

### *In vitro* viral Infections and quantification of viral load

Cells were infected with influenza virus at a multiplicity of infection (MOI) of 5 for 1 h in OptiMEM without trypsin, the inoculum removed by washing, and cells cultured in OptiMEM for 18–24 h. Where indicated, cells were incubated with 10 uM Tpl2 kinase inhibitor (Calbiochem) or equivalent concentration of DMSO vehicle for 30 min prior to infection and throughout infection. RNA, protein, and cell supernatants were collected after 18–24 h. Viral RNA was quantified by RT-PCR using primers specific for influenza virus M gene (Forward 5' CAT GGA ATG GCT AAA GAC AAG ACC, Reverse 5' CCA TTA AGG GCA TTT TGG ACA, Probe FAM- 5' TTT GTG TTC ACG CTC ACC GTG CCC A–TAMRA) and normalized to the level of mouse EF-1 RNA obtained from equivalent volumes of supernatants. To permit comparison between experiments, viral load at 24 h was normalized to initial viral load at 1 h, where indicated. Influenza virus plaque assays were performed by plating serial dilutions of 24 h cell supernatants or BAL fluid on MDCK cells in duplicate, overlaying with agarose, and enumerating crystal violet-stained plaques. Cells were infected with EMCV (MOI = 0.001), VSV (MOI = 0.1) or MCMV (MOI = 10) in complete DMEM for 1 h, cells washed thoroughly to remove inoculum, and incubated for 24 h. EMCV and VSV plaque assays were performed similarly using Vero cells and a methyl cellulose overlay. To adjust for differences in rate of viral replication and cytotoxicity between these 4 viruses, different inoculums were selected to support multiple rounds of infection and replication over 24 h while allowing reproducible quantification of initial infection of the cell monolayer at 1 h.

### Flow cytometry on infected cells

Formalin-fixed cells were permeabilized using 0.2% saponin, stained with anti-influenza NP (ViroStat or Argene) antisera followed by fluorescently conjugated secondary antibodies, and fluorescence measured using a FACSCalibur.

### Western blotting

Western blots were performed on cell lysates by serially incubating membranes with TRAF6 (sc7221, Santa Cruz) or β-actin-HRP (ab20271, Abcam) antibodies, anti-rabbit-HRP secondary antibody, followed by enhanced chemiluminescent detection and exposure to film for various times. Films from non-saturated exposures were quantified by densitometry using Photoshop (Adobe), background subtracted (using pixels contained in an adjacent area of identical size), and TRAF6 pixels/area divided by β-actin pixels/area to give normalized quantification of TRAF6 protein.

### miRNA expression constructs

Constructs expressing both murine mmu-miR-144-3p and mmu-miR-451a were cloned with 145 bp upstream of pre-miR-144 and 330 bp downstream from the end of pre-miR-451. miR-451 alone was expressed with flanking sequences 100 bp upstream and 198 bp downstream. miR-144 was cloned with 145 bp upstream of pre-miR-144 and 198 bp downstream of the end of pre-miR-451; the activity of miR-451 included in this construct was ablated using site-directed mutagenesis of 7 bp of the mature miR-451. These sequences were cloned into retroviral MSCV-GFP or lentiviral pLenti6 (±GFP) vectors. Packaged virus was transduced into the indicated cells, and stably selected using 4 μg/mL puromycin (TC-1 cells) or 2 μg/mL puromycin (LET1 cells). Influenza virus replication phenotypes were confirmed in cells generated by at least 3 independent transductions. LET1 cells were transduced with TRAF6 shRNA lentivirus (sc-36718V, Santa Cruz) or control scrambled shRNA lentivirus (sc-108080V, Santa Cruz) and stably selected using puromycin.

### Luciferase constructs and assays

Luciferase constructs were generated by cloning murine *Irf7* (complete coding sequence +3’-UTR; NM_016850, 1–1830 nt), *Trim30* 3’-UTR, or *Traf6* 3’-UTR (BC060705, 1966–5368 nt; Open Biosystems) 3’ of firefly luciferase. Wild type and mutant miR-144 target sequences in the Traf6 3’-UTR were generated by annealing complementary 45-mer oligos containing the sequences shown in the figure flanked by restriction sites, and cloning directly into the luciferase vector. 293T cells were transfected with pLenti6-miRNA constructs or empty vector along with the UTR-luciferase vector, which gave equivalent results to TC-1 transfections. Cell lysates were collected after 2 d and luciferase luminescence was normalized against a Renilla luciferase control present on the same plasmid (Stop and Glo, Promega).

### Computational analysis

Total RNA was isolated using Trizol from TC-1 cells expressing either MSCV empty vector or MSCV-miR-144+451 and infected with PR8 influenza virus for 24 h. Labeled RNA was hybridized to Affymetrix GeneChip Mouse Exon ST 1.0 arrays, probe-set intensities RMA normalized, and differential expression analysis performed using the Bioconductor package *limma*. Total RNA from LET1 cells was hybridized to Agilent SurePrint G3 Mouse GE 8x60K microarrays. Microarray analysis was performed on 3–5 samples from independent experiments, as indicated in figure legends. Annotated genes with fold changes >2 between conditions were retained for further analysis. Promoter sequences (1 kB upstream of the TSS) were scanned for transcription factor binding-site motifs (TRANSFAC 7.0) using FIMO (MEME suite) with a p-value cut-off of 0.0001. GOMiner was used for GO enrichment determination. miRWalk was used to predict potential miR-144 targets using multiple algorithms (TargetScan, RNA22, PITA, PICTAR4, PICTAR5, RNAHybrid, miRWalk, miRDB, miRanda, and DIANAmT). miRWalk, TargetScan, and RNA22 predicted the miR-144 target sequences in Traf6. The InnateDB curated protein interaction database was used and network visualization was performed using InnateDB and Cytoscape (www.cytoscape.org). Expression data is available at GEO (Accession # GSE31957 (TC-1) and GSE50742 (LET1)).

### Statistical analysis

P-values were determined using an unpaired two-tailed Student’s t-test, assuming equal variances.

## Results

### miR-144 impacts viral replication *in vivo* and in lung epithelial cells infected *in vitro*

**Fig 1:**
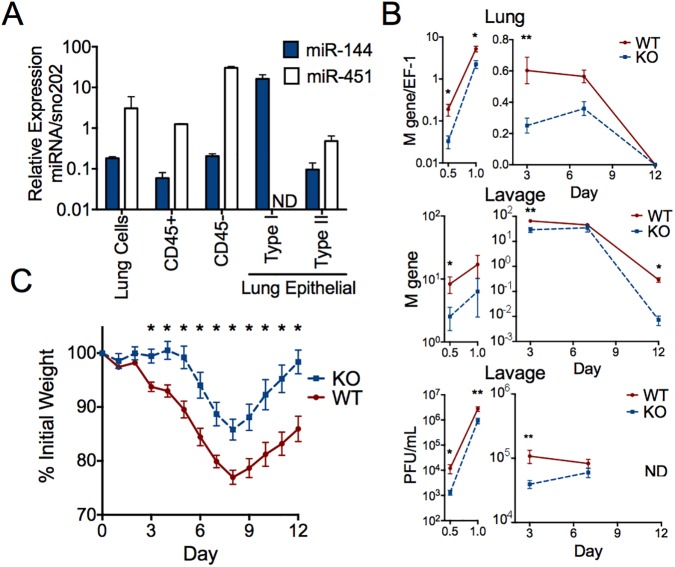
miR-144 and miR-451 are expressed in lung epithelial cells. (*A*) Expression of miR-144 and miR-451 in total lung cells and FACS-purified lung hematopoietic and epithelial cell populations were measured by qRT-PCR and plotted in arbitrary units relative to sno-202 expression. Means ± SEM for 3–8 samples are shown. (*B*) miR-144/451^null^ or wild-type littermate mice were infected with influenza virus for 0.5, 1, 3, 7, or 12 d. Influenza virus was quantified by qRT-PCR of viral M gene in lungs (normalized to EF-1) or BAL, or by plaque assay of BAL. Means ± SEM are shown; 0.5 d (*n* = 8–9), 1 d (*n* = 3–6), 3 d (*n* = 4–11), 7 d (*n* = 5), 12 d (*n* = 3–6). PFUs were below the detection limit at 12 d (ND, not detected). Similar data was obtained in two independent experiments. (*C*) Weight-loss was quantified over 12 d as a correlate of morbidity (*n* = 10–19 mice per time point); reduced weight-loss in KO mice was observed in 5 independent experiments; * p<0.003.

We used the well-characterized murine influenza A virus infection model to investigate how microRNAs may regulate lung epithelial cell responses to respiratory infection and began by globally profiling expression of miRNAs in the influenza virus-infected lung (S1 Table). miR-451 was the most abundant miRNA with an available knockout mouse, which enabled us to functionally test the contribution of this miRNA to host response to influenza virus infection. miR-451 is adjacent to miR-144 on mouse chromosome 11 and both miRNAs are co-expressed in erythroid cells as one transcript that is processed into two mature miRNAs. miR-144 and miR-451 bind to unique sequences in target genes to play non-redundant roles in erythroid lineage differentiation [12, 15, 16] but are not well-characterized in the lung. We used FACS analysis to determine which specific lung cell populations express each of these miRNAs (Fig 1A). miR-144 (mmu-miR-144-3p) expression was much higher in type I lung epithelial cells than in CD45^+^ hematopoietic cells, whereas miR-451 (mmu-miR-451a) was expressed in both hematopoietic and epithelial cells (Fig 1A). Since these miRNAs have been best characterized in cells of the erythroid lineage, we specifically excluded red blood cells from the sorted epithelial cell populations and measured no hemoglobin *Hbb2* expression above the detection limit, excluding the possibility that the miR-144 measured in Ter119^-^ T1α^+^ type I epithelial cells was due contamination by erythroid (Ter119^+^) cells (S2 Table).

We evaluated the effect of miR-144 and miR-451 on viral replication since they are expressed within the natural host cells for influenza [17–19]. miR-144/451-deficient mice exhibit mild anemia due to dysregulated erythroid development and a 2-fold splenic enlargement due to erythroid hyperplasia, but possess an otherwise normal hematopoietic compartment [12, 15, 16]) and normal lung architecture. miR-144/451-deficient mice had decreased viral load in the lung and in bronchoalveolar lavage (BAL) fluid over the first 3 days of infection (Fig 1B), exhibited a delayed onset of weight loss, an established correlate of morbidity and viral load, and recovered their starting weights sooner than wild-type littermates (Fig 1C). Flu-induced lesions were similar in character but decreased in severity and extent in miR-144/451-deficient mice. miR-144/451-deficient mice showed reduced interstitial pneumonia and bronchial epithelial necrosis at day 3, which are characteristics of acute inflammation, and a trend of increased bronchiolar and alveolar epithelial hyperplasia at day 7, which are characteristics of chronic inflammation (S1 and S2 Figs). Alveolar-capillary barrier integrity following infection was equivalent between mice, as indicated by equivalent BAL protein content at early and late time points (S2 Fig). miR-144/451-deficient mice showed increased cellularity in the BAL within the first day of infection and reduced numbers of recruited inflammatory cells at days 3 and 12 (S2 and S3 Figs).

**Fig 2:**
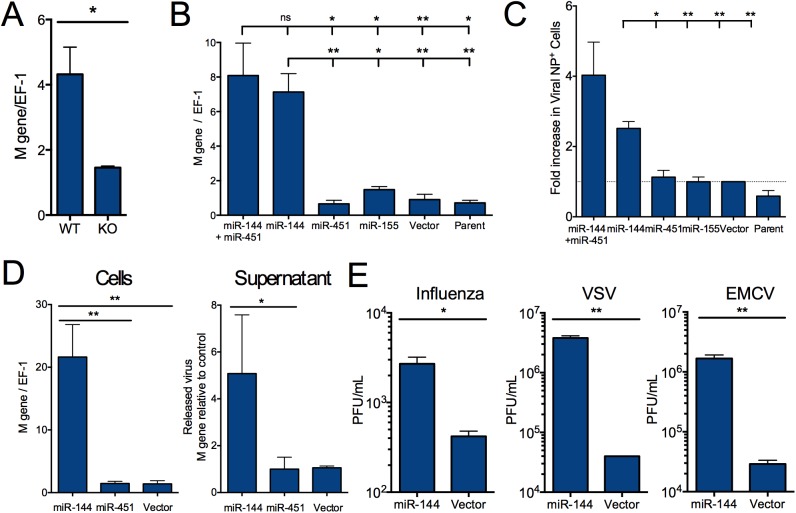
miR-144 impairs control of viral replication by murine lung epithelial cells. (*A*) miR-144/451 KO or WT lung epithelial cells were infected with influenza virus. Mean viral load ±SEM at 24 h was quantified by qRT-PCR of viral M gene and normalized to host EF-1 (*n* = 3). * p<0.05. ** p<0.01, NS, not significant. (*B*) TC-1 cells were stably transduced with retrovirus expressing GFP along with miR-451+miR-144, miR-144, miR-451, miR-155, or retroviral vector alone and compared with untransduced (parent) cells. Viral load in influenza-infected cells was quantified as in *A*. Means ±SEM for 3–7 independent experiments are shown and p-values calculated for miR-144+miR-451 and miR-144-expressing cells relative to each other cell line. * p<0.05. ** p<0.01, NS, not significant. (*C*) TC-1 cells were infected as described in *B* and the mean percentage ±SEM of influenza NP^+^ cells quantified by flow cytometry (*n* = 4–9 independent experiments); p-values were calculated for cells expressing miR-144 alone relative to each other control cell line. (*D*) Primary mouse lung epithelial type I cells (LET1) were immortalized while being transduced with lentivirus expressing miR-144, miR-451, or vector alone. Viral load in influenza-infected cells (*n* = 6–9) or released into supernatants (*n* = 2–7) was measured as described in *B*, and means ±SEM are shown. (*E*) Infectious virus in the supernatants of LET1 cells infected with influenza, EMCV, or VSV measured by plaque assay. Means ±SEM are shown for 3 independent experiments performed in duplicate. A similar increase in viral load was observed when LET1 cells expressing miR-144 were compared with cells expressing miR-451. * p<0.05, ** p<0.01, ns = not significant.

These data suggest that miR-144/miR-451 affects influenza virus replication in epithelial cells, as the largest difference in viral load was observed within the first 12 hours of infection, prior to substantial inflammatory cell recruitment. We have previously observed that lung type I alveolar epithelial cells are rapidly infected in this experimental intranasal infection model, with faster kinetics than the descending infection that occurs during natural infection [20]. We observed a significantly lower viral load in type I epithelial cells isolated from miR-144/451-deficient mice relative to wild-type cells following *in vitro* influenza virus infection (Fig 2A). Obtaining a mechanistic understanding of this *in vivo* phenotype required relevant *in vitro* models for studying influenza virus infection that would permit experimental modulation of miRNA expression. Influenza virus can infect and replicate in multiple types of respiratory epithelial cells; we focused on type I epithelial cells as these are infected by clinically-relevant H1N1 and H3N2 viruses [17, 18, 21] and offered more tractable models than were available for type II epithelial cells, another replicative niche for influenza virus. The expression of miR-144 and miR-451 in three tractable cell culture models is significantly lower than in freshly isolated lung epithelial cells (S4 Fig). Therefore, to develop a model that matches the expression of miR-144/miR-451 *in vivo*, we generated lines of TC-1 and LET1 [14] cells stably over-expressing miR-144, miR-451, miR-155 (as a negative control), or vector alone (S4 Fig). Ectopic expression of miR-144 significantly increased levels of viral genomes and protein (Fig 2B–2E). This increased permissiveness for viral replication was not a generalized response to miRNA expression, since expression of miR-451 or miR-155 did not affect viral replication (Fig 2B and 2C). miR-144 expression significantly increased infectious virion production in cells infected with the negative-sense single-stranded RNA viruses influenza and vesicular stomatitis virus (VSV) and the positive-sense ssRNA encephalomyocarditis virus (EMCV), indicating that the effect of miR-144 is not restricted to influenza virus infection (Fig 2E).

### miR-144 negatively regulates an IRF7-dependent transcriptional network

**Fig 3:**
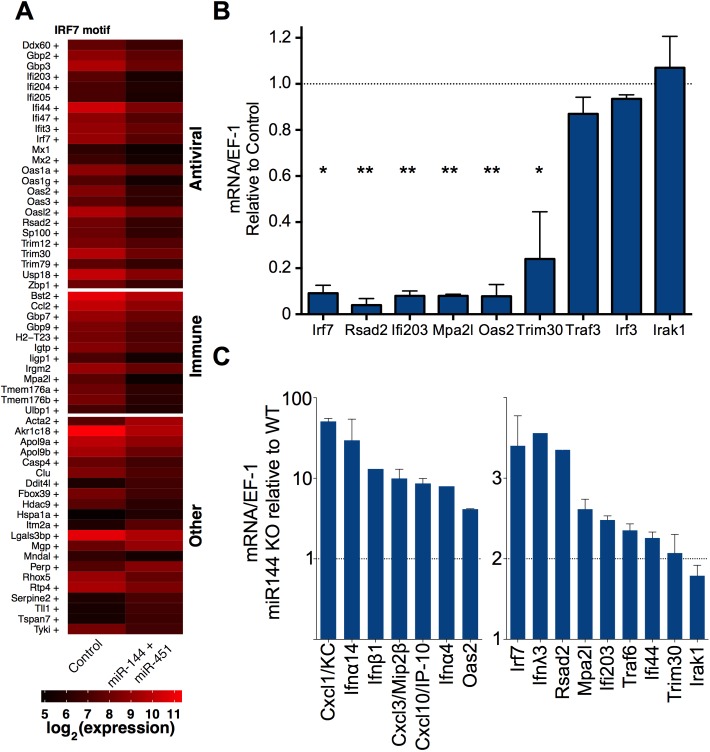
miR-144 attenuates the IRF7 transcriptional network. (*A*) Heat map depicting microarray analysis of influenza-infected TC-1 cells stably expressing miR-144+miR-451 or vector alone, with genes grouped by functional annotation. The indicated genes were differentially expressed by >2-fold after 24 h relative to infected cells expressing vector alone and clustered by biological function. Mean intensities for 3 independent experiments are shown relative to uninfected control (vector alone) cells. Genes containing IRF7 motifs within 1 kB of the transcriptional start site are indicated by “+”. (*B*) Expressions of miR-regulated genes in influenza-infected miR-144+miR-451-expressing TC-1 cells (MOI = 5, 24 h) were measured by qRT-PCR, normalized to EF-1, and plotted relative to the levels in vector-only control cells. Means±SEM for 3 independent experiments are shown. (*C*) qRT-PCR was performed as described in *B* on primary type I epithelial cells sorted from miR-144/451^null^ or WT littermates and infected with influenza virus *in vitro*, *n* = 3. * p<0.05, ** p<0.01.

To elucidate the mechanism whereby miR-144 increases influenza virus replication within lung epithelial cells, we compared the transcriptional profiles of influenza-infected wild-type and miR-144 over-expressing cells. Expression of miR-144 significantly decreased the expression of 48 genes and increased the expression of 9 genes by >2 fold in TC-1 cells (Fig 3A) and similarly impacted the transcriptional profile of LET1 cells (S5 Fig). A dominant feature of the array data was the suppressed expression of genes associated with antiviral and immune interferon responses in cells over-expressing miR-144 (enriched gene ontology (GO) functional categories: defense response to virus and immune response (p<0.05)). A heat map with genes grouped by functional annotations shows two dominant functional modules: antiviral and immune (Fig 3A). We validated the differential expression of several characterized and putative antiviral effectors (*Rsad2*, *Ifi203*, *Mpa2l*, *Oas2*, and *Trim30*) and the transcription factor *Irf7* by qRT-PCR. In contrast, miR-144 expression did not decrease levels of *Irf3*, *Irf1*, or *Irak1*, showing specificity in the transcriptional regulation of a unique subset of antiviral genes (Fig 3B and GSE50742 and GSE31957). The suppressive effect of miR-144 on the expression of this group of antiviral genes was confirmed in LET1 lung epithelial cells ectopically expressing miR-144 (S5A and S5B Fig) and we measured increased expression of this gene module in miR-144^null^ primary type I epithelial cells (Fig 3C).

**Fig 4:**
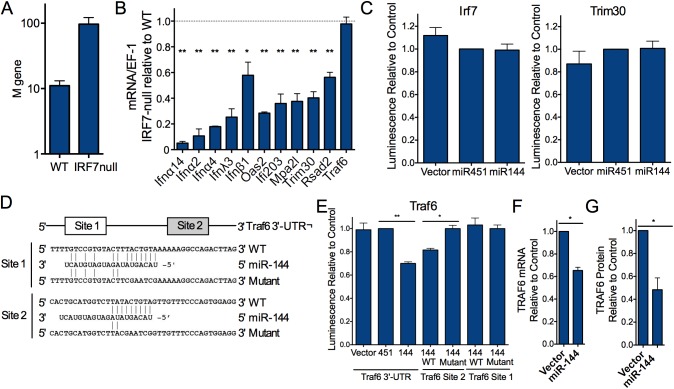
miR-144 negatively regulates IRF7 activity and TRAF6 expression. (*A*) Viral load in the BAL of WT or IRF7^-/-^ mice infected with influenza virus was quantified by qRT-PCR after 24 h. *n* = 3 mice per genotype. (*B*) Gene expression in WT or IRF7^-/-^ mouse lungs following infection with influenza virus for 24 h was measured by qRT-PCR. Mean expression ±SEM in IRF7^-/-^ relative to wild-type mice is plotted. *n* = 3 mice per genotype. (C) Luminescence assay of cells expressing firefly luciferase fused to the complete coding sequence plus 3’-UTR of murine *Irf7* or 3’-UTR only of *Trim30* and miR-144, miR-451, or vector alone. Firefly/Renilla luminescence relative to cells expressing miR-451 for 4 independent experiments performed in triplicate are plotted (means ±SEM). Concordant results were obtained in TC-1 lung epithelial cells. (*D*) Sequences of the 2 computationally predicted miR-144 target sequences in the *Traf6* 3’-UTR and the mutant target sequences used for the experiments shown in *E*. (*E*) Luciferase assays were performed as in *C* using firefly luciferase fused to the complete 3’-UTR of murine *Traf6* or the intact or mutant miR-144 target sites in the *Traf6* 3’-UTR shown in *D*. Means ±SEM for 5 (complete UTR) or 3 (individual miR-144 sites) independent experiments performed in triplicate are shown. *p = 0.014,**p = 0.010. (*F*) Reduced TRAF6 mRNA expression in influenza virus-infected TC-1 cells expressing miR-144 compared to those expressing vector alone by qRT-PCR. Means ±SEM for 3 independent experiments are shown, *p<0.05. (*G*) Reduced TRAF6 protein in TC-1 cells expressing miR-144 compared to those expressing miR-451 or vector alone pre- or 6 h post-infection with influenza virus. See S5D Fig for a representative immunoblot. Means ± SEM for 3 independent experiments are graphically displayed. * p<0.05, ** p<0.01.

We hypothesized a central role for IRF7 in the regulation of the miR-144-regulated transcriptional network because it was the only transcription factor that was differentially expressed and computational predictions identified IRF7 binding motifs within the cis-regulatory elements of the majority of miR-144-regulated genes (Fig 3A). It has been demonstrated previously that IRF7 plays a dominant role in regulating type I and type III IFN-dependent antiviral responses to influenza virus at epithelial surfaces [22–25]. Human IRF7 deficiency results in lower interferon production and impaired control of influenza virus replication [26] and IRF7-null mice are more susceptible to viral infections [22, 27], with increased morbidity and mortality following infection with influenza virus [27]. We observed a 10-fold increase in lung viral load in IRF7-null mice relative to wild type mice after 24 hours (Fig 4A), supporting a role for IRF7-regulated transcriptional programs in the early control of influenza virus infection and concordant with the increased susceptibility of these mice to other viral infections [22, 28, 29].

To identify which of the miR-144-regulated genes are controlled both directly and indirectly by IRF7, we compared gene expression in the lungs of influenza virus-infected IRF7-null and wild type mice. As expected, we observed that the absence of IRF7 significantly reduced the expression of type I and III interferons (*Ifnα2*, *Ifnα4*, *Ifnα14*, *Ifnλ3*, *Ifnβ1*), as well as miR-144-regulated genes in Fig 3 with predicted antiviral functions (*Oas2*, *Ifi203*, *Mpa2l*, *Trim30*, *Rsad2*) (Fig 4B). These data suggest that IRF7 is necessary for normal expression of the network of genes that is regulated by miR-144.

### miR-144 directly regulates TRAF6 expression

We tested whether *Irf7* mRNA is a direct target of miR-144 in a luciferase assay, despite its lack of a computationally predicted miR-144 target sequence. Ectopic expression of miR-144 did not alter expression of a full-length *Irf7*-luciferase construct; thus decreased *Irf7* mRNA levels in miR-144-expressing cells do not result from a direct interaction between *Irf7* and miR-144 (Fig 4C). To establish a potential mechanism for miR-144 inhibiting *Irf7* expression, we used Cytoscape to examine the InnateDB protein-protein interaction database [30] for proteins known to interact directly with IRF7 or with its nearest network neighbors. Computational analysis to identify miR-144 binding sites in the coding and non-coding sequences of the genes that belonged to this expanded network suggested only three candidates where we could hypothesize plausible mechanisms connecting them to our observed viral infection phenotype: *Tpl2/Map3k8*, *Trim30*, and *Traf6*, which each have at least one predicted canonical target sequences in their 3’-UTRs.

TPL2/MAP3K8 is a MAP kinase that can regulate NF-κB and ERK signaling and IFN production [31–33]. We excluded this as a mechanism underlying the observed miR-144 phenotype since ectopic expression miR-144 did not alter Tpl2/Map3K8 expression and chemical inhibition of the kinase did not recapitulate the effect of miR-144 overexpression on viral load (S6A and S6B Fig and GSE50742). TRIM30 is an interferon-induced member of the TRIM family of proteins that can negatively regulate TLR and NLR signaling pathways [34, 35]. Data did not support *Trim30* being a direct target of miR-144 since expression of the 3’-UTR of *Trim30*, *which* contains the predicted miR-144-target sequence, in a luciferase construct was not modulated by miR-144 expression (Fig 4C). TRAF6 is an E3 ubiquitin ligase that is critical for type I interferon responses to viral infection [36]. TRAF6 physically associates with IRF7 [37, 38] and activates its transcriptional activity by mediating K63-linked ubiquitination which permits its subsequent phosphorylation by various kinases [37, 39]. This crucial interaction between TRAF6 and IRF7 is supported by the observation that when IRF7 is mutated so that it cannot be ubiquitinated by TRAF6, it fails to mediate transcriptional responses [40]. We fused the complete 3’-UTR of *Traf6* to firefly luciferase (Fig 4D) and measured a significant decrease in luminescence only in the presence of miR-144 (Fig 4E), to an extent in concordance with published studies for other miRNA-dependent effects [41, 42]. This indicates that miR-144 can post-transcriptionally regulate *Traf6* expression. *Traf6* is predicted to contain two miR-144 target sequences in its 3’-UTR. We generated luciferase constructs containing individual intact or mutated miR-144 target sites (Fig 4D). Mutation of 7 nucleotides in the predicted miR-144 target sequence in site 2 completely abrogated the suppressive effect of miR-144 on expression of the reporter construct, while mutating the same nucleotides in site 1 had no effect (Fig 4E). We observed a significantly reduced level of *Traf6* mRNA and protein in cells expressing miR-144 (Figs 4F and 4G, S5C and S5D) and higher TRAF6 mRNA in miR-144-deficient cells (Fig 3C). TRAF6 has also been shown to be regulated by miR-146a [43, 44]; however, miR-144 expression did not alter miR-146a expression in cells (S6C Fig), and therefore we have no data to suggest that miR-144 regulates TRAF6 via miR-146a. Together, while miR-144 may interact with other target mRNAs to contribute to this antiviral phenotype, these luciferase data and quantification of TRAF6 mRNA and protein abundance indicate that miR-144 can negatively regulate TRAF6 levels.

### Decreased TRAF6 levels are sufficient to impair host control of influenza virus replication

**Fig 5:**
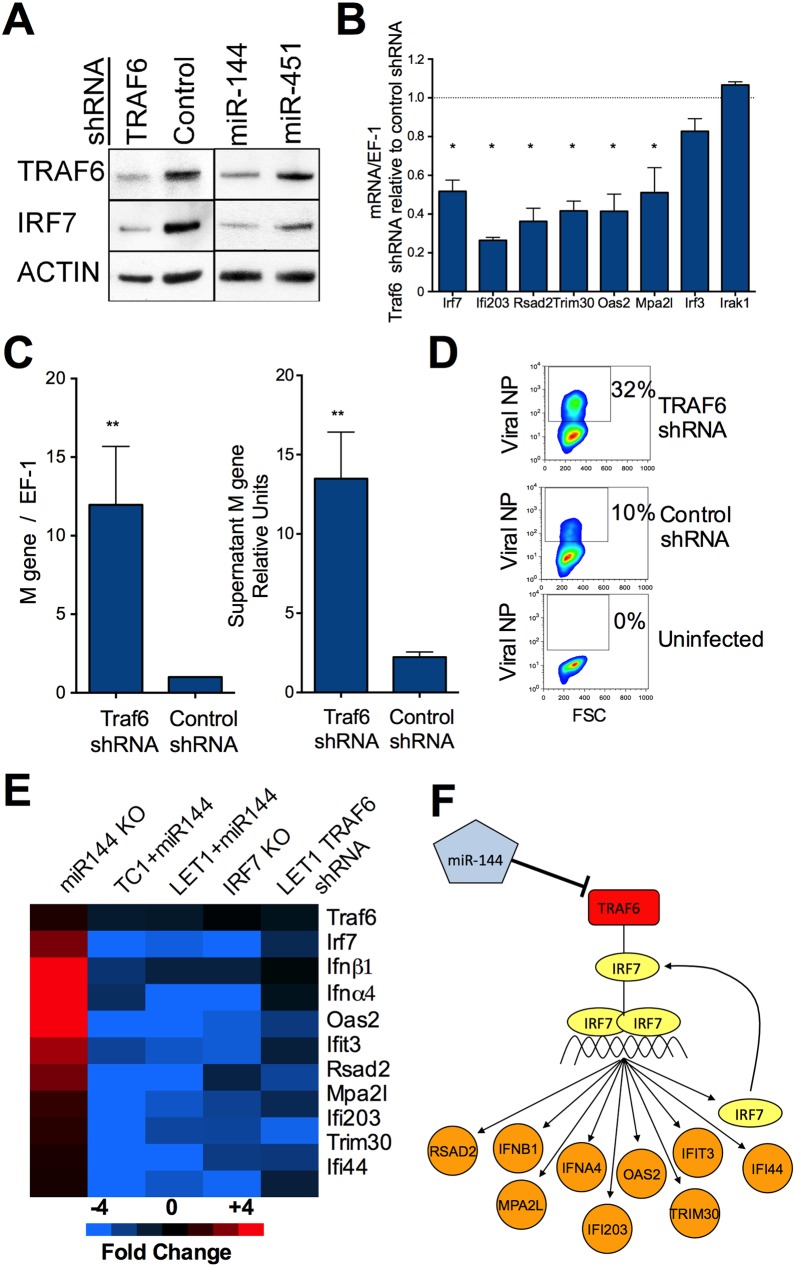
Decreased TRAF6 levels are sufficient to impair host control of influenza virus replication. (*A*) Western blotting of TRAF6, IRF7, and β-actin was performed using protein lysates from the indicated LET1 cells. Densitometric quantification of 3–4 independent experiments was performed and means ±SEM normalized to actin and relative to control shRNA cells is as follows: TRAF6 Western. Control shRNA, 1; TRAF6 shRNA, 0.43±0.15; miR-144, 0.52±0.12; miR-451, 0.79±0.12. IRF7 Western. Control shRNA, 1; TRAF6 shRNA, 0.47±0.12; miR-144, 0.68±0.09; miR-451, 1.16±0.26. (*B*) Gene expression by qRT-PCR in LET1 cells stably expressing TRAF6-specific shRNAs and infected with influenza virus for 18 h is shown relative to the level in infected LET1 cells expressing control shRNA. Means ±SEM for 3 independent experiments performed in duplicate are shown. (*C*) qRT-PCR of influenza viral load in LET1 cells expressing TRAF6-specific or control shRNAs or in the cell-free supernatants 18 h post-infection. Means ± SEM for 3 independent experiments performed in duplicate are shown. MOI = 5; * p<0.05, ** p<0.01. (*D*) Cells infected as described in *C* were stained for viral NP protein and analyzed by flow cytometry. Data are representative of 2 independent experiments. (*E*) Heat map of antiviral gene expression generated from the log_2_ ratios of gene expression between the following cells: miR-144^-/-^/miR-144^+/+^ type I lung epithelial cells, TC-1+miR-144+miR-451/TC-1+vector alone, LET1+miR-144/LET1+vector alone, IRF7null vs WT lungs, LET1+TRAF6 shRNAs/LET1+control shRNA. All cells were infected with PR8 influenza virus for 24 h, gene expression measured by qRT-PCR, and mean intensities for 3 independent samples compared. Red (blue) represents up-(down-) regulation relative to controls. (*F*) Network model for miR-144 regulation of the TRAF6-IRF7-antiviral network.

As TRAF6-dependent K63-ubiquitination of IRF7 is necessary for its transcriptional activity [40], our data support a model whereby miR-144 modulates the expression of a network of IRF7-regulated genes by targeting the 3’-UTR of Traf6 mRNA. We hypothesized that cells expressing shRNAs specific for *Traf6* should have a similarly impaired antiviral capacity as cells expressing miR-144 if reduced steady state TRAF6 levels are mechanistically linked to impaired IRF7-dependent transcription. To test this hypothesis, we generated immortalized lung type I epithelial cells with TRAF6 protein levels reduced by miR-144 or specific shRNAs, along with control cells expressing miR-451 or a non-functional shRNA (Fig 5A). Reduced TRAF6 levels were associated with reduced IRF7 protein and mRNA levels (Fig 5A and 5B), which is expected as IRF7 positively feedbacks on its own transcription to amplify the IRF7-dependent transcriptional program [22]. These decreased levels of IRF7 have functional consequences for the IRF7 network. For example, expression of *Ifi203*, *Rsad2*, *Trim30*, *Oas2*, and *Mpa2l* is impaired when TRAF6 levels are suppressed by specific shRNA (Fig 5B) or miR-144 (Figs 3B and S5B). The role of TRAF6 as the proximal component in the TRAF6-IRF7-IFN antiviral network is further supported by the observation that decreased TRAF6 levels brought about by two independent approaches (overexpressed miR-144 or anti-TRAF6 shRNA) are associated with significantly increased viral replication (Fig 2 and Fig 5C and 5D, respectively). We observe concordant decreased expression of a module of antiviral genes in cells over-expressing miR-144, TRAF6 shRNAs, and IRF7-deficient cells (Fig 5E, blue), and reciprocal expression in miR-144-deficient cells (Fig 5E red). These data support the network model depicted in Fig 5F, where miR-144 suppresses expression of the TRAF6-IRF7-IFN-regulated gene expression network to diminish the antiviral capacity of influenza virus-infected cells.

## Discussion

We have demonstrated microRNA attenuation of the host antiviral response using a miRNA knockout mouse and *in vitro* models. Reciprocal data obtained from gain- and loss-of-function studies shows that miR-144 modulates an antiviral transcriptional network within lung epithelial cells. The predominant effect of miR-144 deficiency was to decrease viral load rather than modulate inflammatory responses within the virus-infected lung, and better control of very early viral replication within epithelial cells significantly decreased morbidity. We employed primary lung epithelial cells and cell lines since they are a relevant replicative niche for influenza virus, and ectopically expressed miR-144 at physiological levels as a strategy to identify biologically relevant targets of miR-144. miR-144 also increased virion production following infection with VSV, a negative-sense single-stranded RNA viruses, and EMCV, a positive-sense ssRNA encephalomyocarditis virus, indicating that the effect of miR-144 is not restricted to influenza virus.

Recent studies have identified miRNAs with proviral effects in influenza virus-infected cells, resulting in increased viral replication [45, 46], while other miRNAs impair viral replication [47]. mRNAs targeted by miRNAs in influenza-infected cells encode a range of proteins relevant to the immune response to infection, including viral sensors [46], cytokines [48, 49], and interferon [50]. Global proteomics studies have shown that miRNAs modestly decrease protein levels of many targets. Therefore, while decreasing TRAF6 levels by a similar extent through expression of miR-144 or TRAF6 shRNAs was sufficient to impair the antiviral capacity of epithelial cells, it is probable that miR-144 also regulates other target genes to mediate this cellular phenotype. One caveat of our study is that it utilized transcriptional profiling of miR-144 overexpressing cells to identify potential miR-144 target genes, which will not identify miRNA-mRNA interactions that impact protein translation. It is also relevant to consider potential interplay between miRNAs regulating the same pathway: miR-146a also targets TRAF6 and is induced following infection [43, 44, 51] and therefore could play a more prominent role in negatively regulating TRAF6-dependent antiviral responses at later stages of infection. Negative regulation of multiple genes in a signaling pathway or of a single gene by multiple miRNAs could explain how modest effects on individual targets can result in robust phenotypes.

The relevance of miRNAs in regulating viral replication has been questioned by data from Dicer-deficient 293T cells that lack nearly all miRNAs, which showed normal replication by a large number of clinically-relevant viruses, including influenza A virus [52]. Aguado et al. employed a rapid vector-mediated miRNA ablation system and showed that cellular miRNAs predominantly regulate cytokine rather than antiviral effector genes in 293T and human foreskin fibroblast cells during acute viral infection [53]. In contrast, we observe a significant impact of the loss of the miR-144/451 locus on lung viral load rather than cytokines (Fig 1). We measure expression levels of miR-144 that is four orders of magnitude higher in primary normal human bronchial epithelial cells, the host cell targeted by influenza A virus, compared with the 293T kidney cell line (S4 Fig). Therefore, the impact we observe of miR-144 on influenza virus replication in lung epithelial cells could not be modeled by the *in vitro* system employed in those two studies. We interpret these data as additional support for the emerging appreciation of the contextual nature of miRNA-target interactions.

Mice deficient in miR-144 showed improved control of influenza virus replication, leading us to speculate that constitutive expression of this negative regulatory mechanism must not be deleterious and could be advantageous in some contexts. Appropriate regulation of epithelial responses during viral infection is critical to sufficiently activate effector functions that limit viral replication while minimizing tissue damage [54]. Seo et al demonstrated that miRNAs negatively regulate many interferon-stimulated genes in uninfected cells, particularly those associated with cell death and proliferation, in order to maintain homeostasis [55]. Low basal IFN-α expression has been suggested to set an activation threshold by priming cells to respond more rapidly and robustly to viral infection, in part by regulating IRF7 levels [23]. Negative regulation of TRAF6 levels by miR-144 provides an additional layer of post-transcriptional control of IRF7. miRNAs can exert cell type-specific roles based on restriction of miRNA expression to specific cell types or differing relative abundance of mRNA target sequences. Plasmacytoid dendritic cells are specialized rapid and robust producers of type I interferons. miR-144 is not detected in plasmacytoid dendritic cells [48, 56] suggesting that our observations of the effect of miR-144 in lung epithelial cells do not extend to regulation of basal IRF7-dependent antiviral gene expression in plasmacytoid dendritic cells. Cell lineage-specific expression of negative regulators in mucosal tissues, such as the lung, provides a potential mechanism for setting a higher threshold of immune activation than that required at sterile sites. Constitutive expression of a different microRNA was shown to contribute to the establishment of mucosal tolerance within the neonatal intestine [57], lending support to a model where basal miRNA expression at mucosal surfaces counterbalances immune transcriptional activation from environmental insults with post-transcriptional repression.

## Supporting information

S1 Table MicroRNA profiling of lungs from C57Bl/6 mice infected with Influenza PR8 for 72 h. Data is shown normalized to the housekeeping small RNAs sno-202 or U6.
(XLS)

S2 Table Expression levels of Hbb2, a marker of contamination by erythroid cells, is not detected above the detection limit of 35 qRT-PCR cycles in sorted type I epithelial cells. Cycle thresholds (Ct) for qRT-PCR using Hbb2-specific primers and probe on independent whole lung containing red blood cells or FACS purified Ter119^-^ T1α^+^ type I epithelial cells are shown.
(XLS)

S1 Fig **Histology of miR-144/451**^**-/-**^ **lungs infected with influenza virus.** (A) Representative hematoxylin and eosin-stained lung sections from wild type and miR-144/451^-/-^ mice as indicated. Infection with PR8 (700 pfu) as indicated with D0 representing uninfected controls for comparison of normal morphology in both genotypes. Low power overviews (upper row scale bars = 8mm for all) demonstrate regional distribution of lesions (darker consolidated areas) with decreased extent in the miR-144/451^-/-^ sections. Higher magnifications (upper row scale bars = 400μm for all) correspond to boxed regions within low power overviews. Influenza virus-induced lesions are similar in character but are decreased in severity or extent in miR-144/451^-/-^ with both genotypes demonstrating acute and chronic changes. (B) Representative example of scored acute and chronic changes graphed in Fig 1D (scale bar = 400μm). Acute changes scored include necrosuppurative bronchiolitis (here with regional interstitial spread) and perivascular neutrophils. Other acute lesions in this example from a WT mouse at d3 include intrabronchial necrotic debris, perivascular edema, minimal hemorrhage, and vascular lesions (marginating inflammatory cells, reactive endothelia). Chronic lesions scored included bronchiolar and alveolar hyperplasia, perivascular mononuclear cells and lymphoid aggregates. Other chronic lesions noted in this example from a miR-144/451^-/-^ d12 mouse include mild goblet cell hyperplasia in the large airway and diffuse lymphocytic interstitial pneumonia and alveolitis with mild hemorrhage.
(TIF)

S2 Fig Impact of miR-144 deficiency on histopathology and inflammatory cell infiltration during influenza virus infection. (*A*) Mice were infected with influenza virus for 3 or 12 d as described in Fig 1. Mean histological scores are shown (scales of 1–4 each for severity and extent of section affected in the most severe manner). Pneumonia = interstitial pneumonia/alveolitis x extent of the most severe changes; Bronchial necrosis = necrotizing bronchiolitis x extent of the most severe changes; Acute inflammation = necrosuppurative bronchiolitis + perivascular neutrophils; Chronic inflammation = bronchial hyperplasia + alveolar hyperplasia + perivascular mononuclear cells + lymphoid aggregates. 3 d: *n* = 13–14, 7 d, *n* = 4–6, 12 d: *n* = 4–6. (*B*) Protein content as a surrogate metric of lung barrier function was quantified by BCA assay of BAL fluid obtained from 4–14 mice per time point; p>0.1 for all time points. (*C*) The number of inflammatory cells obtained in the BAL was enumerated and means ±SEM (*n* = 4–6) are representative of 1–3 independent experiments.
(TIFF)

S3 Fig miR-144 deficiency affects specific populations of cells infiltrating the lung following influenza virus infection. Cells collected by bronchoalveolar lavage or enzymatic dissociation of infected lung tissue were stained with a panel of cell lineage-specific antibodies and analyzed by flow cytometry. Medians are plotted; * p<0.05.
(TIF)

S4 Fig Generation of an *in vitro* model to study the mechanism of miR-144’s effect on host antiviral response. Expression of miR-144 and miR-451 in primary type I lung epithelial cells was compared to the expression level in primary polarized tracheal epithelial cells (mTEC), cultured primary lung alveolar epithelial type I cells (LET1), mouse TC-1 epithelial cell lines with or without stable transduction of microRNAs, and 293T cells. Expression was measured by qRT-PCR and plotted relative to sno-202 expression. Means ±SEM are shown for 3–8 cellular samples. ND = not determined.
(TIFF)

S5 Fig miR-144 regulates the IRF7 transcriptional network in LET1 cells. (*A*) Microarray transcriptional analysis of LET1 cells stably expressing miR-144, miR-451, or vector alone were infected with influenza virus for 1 h. The heatmap depicts fold-change relative to a vector control for the set of genes whose expression in TC-1 cells following influenza virus infection was affected more than 2-fold (p<0.05) by miR-144/451 over-expression (Fig 3A) with red and green representing up- and down-regulation respectively. Mean relative intensities for 5 (miR-144) or 2 (miR-451) independent experiments using 5 (vector alone) control samples are shown. Complete expression data is available at GEO (GSE50742). (*B*) qRT-PCR was performed as described in Fig 3*B* on influenza-infected LET1 cells, with gene expression in cells stably expressing miR-144 alone shown relative to cells expressing vector alone; *n* = 2 and representative of 3 experiments. (*C*) TRAF6 expression in the cells described in *A* was measured by Agilent microarray. Means ± SEM are plotted for *n* = 5 (miR-144 and vector) or *n* = 2 (miR-451); *p = 0.013. (*D*) Reduced TRAF6 protein in TC-1 cells expressing miR-144 compared to those expressing miR-451 or vector alone pre- or 6 h post-infection with influenza virus. TRAF6 protein expression relative to actin was quantified by densitometry of Western blots: Uninfected: miR-451, 0.70; miR-144, 0.37; Infected: miR-451: 1.0; miR-144: 0.66. Representative Western blot for the 3 experiments graphically displayed in Fig 4G.
(TIF)

S6 Fig No evidence for miR-144 affecting Tpl2/Map3K8 or miR-146a expression. (A) Ectopic expression of miR-144 in TC-1 or LET1 cells did not alter Tpl2/Map3K8 expression. qRT-PCR gene expression normalized by EF-1 levels and plotted in arbitrary units; means ±SEM (*n* = 2–10). (B) Chemical inhibition of the Tpl2 kinase did not increase influenza virus replication over 24 h in LET1 cells overexpressing miR-144 or miR-451 (control), as assessed by qRT-PCR of M gene normalize by EF-1; means ±SEM (*n* = 3–4). (C) Expression of miR-146a is equivalent in LET1 cells expressing miR-144 compared with cells expressing miR-451 as a control, or vector alone. miR-146a measured by qRT-PCR is plotted in arbitrary units relative to U6 expression. Means ± SEM for 2–4 samples are shown.
(TIFF)

## References

[1] Trinchieri G. Type I interferon: friend or foe? J Exp Med. 2010;207(10):2053–63. Epub 2010/09/15. doi: 10.1084/jem.20101664 20837696 10.1084/jem.20101664 PMC2947062

[2] Ehrhardt C, Seyer R, Hrincius ER, Eierhoff T, Wolff T, Ludwig S. Interplay between influenza A virus and the innate immune signaling. Microbes Infect. 2010;12(1):81–7. Epub 2009/09/29. doi: 10.1016/j.micinf.2009.09.007 19782761 10.1016/j.micinf.2009.09.007

[3] Sadler AJ, Williams BR. Interferon-inducible antiviral effectors. Nat Rev Immunol. 2008;8(7):559–68. Epub 2008/06/26. doi: 10.1038/nri2314 18575461 10.1038/nri2314 PMC2522268

[4] Bartel DP. MicroRNAs: target recognition and regulatory functions. Cell. 2009;136(2):215–33. Epub 2009/01/27. doi: 10.1016/j.cell.2009.01.002 19167326 10.1016/j.cell.2009.01.002 PMC3794896

[5] Na YJ, Kim JH. Understanding cooperativity of microRNAs via microRNA association networks. BMC Genomics. 2013;14 Suppl 5:S17. PubMed Central PMCID: PMCPMC3852507. 24552551 10.1186/1471-2164-14-S5-S17 PMC3852507

[6] Mehta A, Baltimore D. MicroRNAs as regulatory elements in immune system logic. Nat Rev Immunol. 2016;16(5):279–94. doi: 10.1038/nri.2016.40 27121651 10.1038/nri.2016.40

[7] Xiao C, Rajewsky K. MicroRNA control in the immune system: basic principles. Cell. 2009;136(1):26–36. Epub 2009/01/13. doi: 10.1016/j.cell.2008.12.027 19135886 10.1016/j.cell.2008.12.027

[8] Skalsky RL, Cullen BR. Viruses, microRNAs, and Host Interactions. Annu Rev Microbiol. 2010;64:123–41. Epub 2010/05/19. doi: 10.1146/annurev.micro.112408.134243 20477536 10.1146/annurev.micro.112408.134243 PMC3621958

[9] Globinska A, Pawelczyk M, Kowalski ML. MicroRNAs and the immune response to respiratory virus infections. Expert Rev Clin Immunol. 2014;10(7):963–71. doi: 10.1586/1744666X.2014.913482 24784476 10.1586/1744666X.2014.913482

[10] Jopling CL, Yi M, Lancaster AM, Lemon SM, Sarnow P. Modulation of hepatitis C virus RNA abundance by a liver-specific MicroRNA. Science. 2005;309(5740):1577–81. Epub 2005/09/06. doi: 10.1126/science.1113329 16141076 10.1126/science.1113329

[11] Henke JI, Goergen D, Zheng J, Song Y, Schuttler CG, Fehr C, et al microRNA-122 stimulates translation of hepatitis C virus RNA. EMBO J. 2008;27(24):3300–10. Epub 2008/11/21. doi: 10.1038/emboj.2008.244 19020517 10.1038/emboj.2008.244 PMC2586803

[12] Yu D, dos Santos CO, Zhao G, Jiang J, Amigo JD, Khandros E, et al miR-451 protects against erythroid oxidant stress by repressing 14-3-3zeta. Genes Dev. 2010;24(15):1620–33. Epub 2010/08/04. doi: 10.1101/gad.1942110 20679398 10.1101/gad.1942110 PMC2912560

[13] Herold S, von Wulffen W, Steinmueller M, Pleschka S, Kuziel WA, Mack M, et al Alveolar epithelial cells direct monocyte transepithelial migration upon influenza virus infection: impact of chemokines and adhesion molecules. J Immunol. 2006;177(3):1817–24. Epub 2006/07/20. 16849492 10.4049/jimmunol.177.3.1817

[14] Rosenberger CM, Podyminogin RL, Askovich PS, Navarro G, Kaiser SM, Sanders CJ, et al Characterization of innate responses to influenza virus infection in a novel lung type I epithelial cell model. J Gen Virol. 2014;95(Pt 2):350–62. Epub 2013/11/19. PubMed Central PMCID: PMC3917066. doi: 10.1099/vir.0.058438-0 24243730 10.1099/vir.0.058438-0 PMC3917066

[15] Rasmussen KD, Simmini S, Abreu-Goodger C, Bartonicek N, Di Giacomo M, Bilbao-Cortes D, et al The miR-144/451 locus is required for erythroid homeostasis. J Exp Med. 2010;207(7):1351–8. Epub 2010/06/02. doi: 10.1084/jem.20100458 20513743 10.1084/jem.20100458 PMC2901075

[16] Papapetrou EP, Korkola JE, Sadelain M. A genetic strategy for single and combinatorial analysis of miRNA function in mammalian hematopoietic stem cells. Stem Cells. 2010;28(2):287–96. Epub 2009/11/17. doi: 10.1002/stem.257 19911427 10.1002/stem.257

[17] Shieh WJ, Blau DM, Denison AM, Deleon-Carnes M, Adem P, Bhatnagar J, et al 2009 pandemic influenza A (H1N1): pathology and pathogenesis of 100 fatal cases in the United States. Am J Pathol. 2010;177(1):166–75. Epub 2010/05/29. doi: 10.2353/ajpath.2010.100115 20508031 10.2353/ajpath.2010.100115 PMC2893660

[18] Chan MC, Chan RW, Yu WC, Ho CC, Yuen KM, Fong JH, et al Tropism and innate host responses of the 2009 pandemic H1N1 influenza virus in ex vivo and in vitro cultures of human conjunctiva and respiratory tract. Am J Pathol. 2010;176(4):1828–40. Epub 2010/01/30. doi: 10.2353/ajpath.2010.091087 20110407 10.2353/ajpath.2010.091087 PMC2843473

[19] Sanders CJ, Doherty PC, Thomas PG. Respiratory epithelial cells in innate immunity to influenza virus infection. Cell Tissue Res. 2011;343(1):13–21. Epub 2010/09/18. doi: 10.1007/s00441-010-1043-z 20848130 10.1007/s00441-010-1043-z

[20] Askovich PS, Sanders CJ, Rosenberger CM, Diercks AH, Dash P, Navarro G, et al Differential host response, rather than early viral replication efficiency, correlates with pathogenicity caused by influenza viruses. PLoS One. 2013;8(9):e74863 PubMed Central PMCID: PMCPMC3779241. doi: 10.1371/journal.pone.0074863 24073225 10.1371/journal.pone.0074863 PMC3779241

[21] van Riel D, Munster VJ, de Wit E, Rimmelzwaan GF, Fouchier RA, Osterhaus AD, et al Human and avian influenza viruses target different cells in the lower respiratory tract of humans and other mammals. Am J Pathol. 2007;171(4):1215–23. PubMed Central PMCID: PMCPMC1988871. doi: 10.2353/ajpath.2007.070248 17717141 10.2353/ajpath.2007.070248 PMC1988871

[22] Honda K, Yanai H, Negishi H, Asagiri M, Sato M, Mizutani T, et al IRF-7 is the master regulator of type-I interferon-dependent immune responses. Nature. 2005;434(7034):772–7. Epub 2005/04/01. doi: 10.1038/nature03464 15800576 10.1038/nature03464

[23] Sato M, Suemori H, Hata N, Asagiri M, Ogasawara K, Nakao K, et al Distinct and essential roles of transcription factors IRF-3 and IRF-7 in response to viruses for IFN-alpha/beta gene induction. Immunity. 2000;13(4):539–48. Epub 2000/11/09. 11070172 10.1016/s1074-7613(00)00053-4

[24] Marie I, Durbin JE, Levy DE. Differential viral induction of distinct interferon-alpha genes by positive feedback through interferon regulatory factor-7. EMBO J. 1998;17(22):6660–9. Epub 1998/11/21. doi: 10.1093/emboj/17.22.6660 9822609 10.1093/emboj/17.22.6660 PMC1171011

[25] Crotta S, Davidson S, Mahlakoiv T, Desmet CJ, Buckwalter MR, Albert ML, et al Type I and type III interferons drive redundant amplification loops to induce a transcriptional signature in influenza-infected airway epithelia. PLoS pathogens. 2013;9(11):e1003773 Epub 2013/11/28. PubMed Central PMCID: PMC3836735. doi: 10.1371/journal.ppat.1003773 24278020 10.1371/journal.ppat.1003773 PMC3836735

[26] Ciancanelli MJ, Huang SX, Luthra P, Garner H, Itan Y, Volpi S, et al Infectious disease. Life-threatening influenza and impaired interferon amplification in human IRF7 deficiency. Science. 2015;348(6233):448–53. PubMed Central PMCID: PMCPMC4431581. doi: 10.1126/science.aaa1578 25814066 10.1126/science.aaa1578 PMC4431581

[27] Hatesuer B, Hoang HT, Riese P, Trittel S, Gerhauser I, Elbahesh H, et al Deletion of Irf3 and Irf7 Genes in Mice Results in Altered Interferon Pathway Activation and Granulocyte-Dominated Inflammatory Responses to Influenza A Infection. J Innate Immun. 2016. 10.1159/000450705 PMC6738875 27811478

[28] Steinberg C, Eisenacher K, Gross O, Reindl W, Schmitz F, Ruland J, et al The IFN regulatory factor 7-dependent type I IFN response is not essential for early resistance against murine cytomegalovirus infection. Eur J Immunol. 2009;39(4):1007–18. Epub 2009/03/14. doi: 10.1002/eji.200838814 19283778 10.1002/eji.200838814

[29] Lang PA, Cervantes-Barragan L, Verschoor A, Navarini AA, Recher M, Pellegrini M, et al Hematopoietic cell-derived interferon controls viral replication and virus-induced disease. Blood. 2009;113(5):1045–52. Epub 2008/10/31. doi: 10.1182/blood-2007-10-117861 18971424 10.1182/blood-2007-10-117861

[30] Lynn DJ, Winsor GL, Chan C, Richard N, Laird MR, Barsky A, et al InnateDB: facilitating systems-level analyses of the mammalian innate immune response. Molecular systems biology. 2008;4:218 Epub 2008/09/04. PubMed Central PMCID: PMC2564732. doi: 10.1038/msb.2008.55 18766178 10.1038/msb.2008.55 PMC2564732

[31] Yang HT, Papoutsopoulou S, Belich M, Brender C, Janzen J, Gantke T, et al Coordinate regulation of TPL-2 and NF-kappaB signaling in macrophages by NF-kappaB1 p105. Molecular and cellular biology. 2012;32(17):3438–51. Epub 2012/06/27. PubMed Central PMCID: PMC3422004. doi: 10.1128/MCB.00564-12 22733995 10.1128/MCB.00564-12 PMC3422004

[32] Dumitru CD, Ceci JD, Tsatsanis C, Kontoyiannis D, Stamatakis K, Lin JH, et al TNF-alpha induction by LPS is regulated posttranscriptionally via a Tpl2/ERK-dependent pathway. Cell. 2000;103(7):1071–83. Epub 2001/02/13. 11163183 10.1016/s0092-8674(00)00210-5

[33] Xiao N, Eidenschenk C, Krebs P, Brandl K, Blasius AL, Xia Y, et al The Tpl2 mutation Sluggish impairs type I IFN production and increases susceptibility to group B streptococcal disease. J Immunol. 2009;183(12):7975–83. Epub 2009/11/20. doi: 10.4049/jimmunol.0902718 19923465 10.4049/jimmunol.0902718 PMC8424661

[34] Shi M, Deng W, Bi E, Mao K, Ji Y, Lin G, et al TRIM30 alpha negatively regulates TLR-mediated NF-kappa B activation by targeting TAB2 and TAB3 for degradation. Nature immunology. 2008;9(4):369–77. Epub 2008/03/18. doi: 10.1038/ni1577 18345001 10.1038/ni1577

[35] Hu Y, Mao K, Zeng Y, Chen S, Tao Z, Yang C, et al Tripartite-motif protein 30 negatively regulates NLRP3 inflammasome activation by modulating reactive oxygen species production. J Immunol. 2010;185(12):7699–705. Epub 2010/11/05. doi: 10.4049/jimmunol.1001099 21048113 10.4049/jimmunol.1001099

[36] Yoshida R, Takaesu G, Yoshida H, Okamoto F, Yoshioka T, Choi Y, et al TRAF6 and MEKK1 play a pivotal role in the RIG-I-like helicase antiviral pathway. J Biol Chem. 2008;283(52):36211–20. Epub 2008/11/06. doi: 10.1074/jbc.M806576200 18984593 10.1074/jbc.M806576200 PMC2662295

[37] Kawai T, Sato S, Ishii KJ, Coban C, Hemmi H, Yamamoto M, et al Interferon-alpha induction through Toll-like receptors involves a direct interaction of IRF7 with MyD88 and TRAF6. Nat Immunol. 2004;5(10):1061–8. Epub 2004/09/14. doi: 10.1038/ni1118 15361868 10.1038/ni1118

[38] Honda K, Yanai H, Mizutani T, Negishi H, Shimada N, Suzuki N, et al Role of a transductional-transcriptional processor complex involving MyD88 and IRF-7 in Toll-like receptor signaling. Proc Natl Acad Sci U S A. 2004;101(43):15416–21. Epub 2004/10/20. doi: 10.1073/pnas.0406933101 15492225 10.1073/pnas.0406933101 PMC523464

[39] Konno H, Yamamoto T, Yamazaki K, Gohda J, Akiyama T, Semba K, et al TRAF6 establishes innate immune responses by activating NF-kappaB and IRF7 upon sensing cytosolic viral RNA and DNA. PLoS One. 2009;4(5):e5674 Epub 2009/05/30. doi: 10.1371/journal.pone.0005674 19479062 10.1371/journal.pone.0005674 PMC2682567

[40] Ning S, Campos AD, Darnay BG, Bentz GL, Pagano JS. TRAF6 and the three C-terminal lysine sites on IRF7 are required for its ubiquitination-mediated activation by the tumor necrosis factor receptor family member latent membrane protein 1. Mol Cell Biol. 2008;28(20):6536–46. Epub 2008/08/20. doi: 10.1128/MCB.00785-08 18710948 10.1128/MCB.00785-08 PMC2577435

[41] Skalsky RL, Corcoran DL, Gottwein E, Frank CL, Kang D, Hafner M, et al The viral and cellular microRNA targetome in lymphoblastoid cell lines. PLoS Pathog. 2012;8(1):e1002484 PubMed Central PMCID: PMCPMC3266933. doi: 10.1371/journal.ppat.1002484 22291592 10.1371/journal.ppat.1002484 PMC3266933

[42] Mehta A, Zhao JL, Sinha N, Marinov GK, Mann M, Kowalczyk MS, et al The MicroRNA-132 and MicroRNA-212 Cluster Regulates Hematopoietic Stem Cell Maintenance and Survival with Age by Buffering FOXO3 Expression. Immunity. 2015;42(6):1021–32. PubMed Central PMCID: PMCPMC4471877. doi: 10.1016/j.immuni.2015.05.017 26084022 10.1016/j.immuni.2015.05.017 PMC4471877

[43] Taganov KD, Boldin MP, Chang KJ, Baltimore D. NF-kappaB-dependent induction of microRNA miR-146, an inhibitor targeted to signaling proteins of innate immune responses. Proc Natl Acad Sci U S A. 2006;103(33):12481–6. Epub 2006/08/04. doi: 10.1073/pnas.0605298103 16885212 10.1073/pnas.0605298103 PMC1567904

[44] Hou J, Wang P, Lin L, Liu X, Ma F, An H, et al MicroRNA-146a feedback inhibits RIG-I-dependent Type I IFN production in macrophages by targeting TRAF6, IRAK1, and IRAK2. J Immunol. 2009;183(3):2150–8. Epub 2009/07/15. doi: 10.4049/jimmunol.0900707 19596990 10.4049/jimmunol.0900707

[45] Wolf S, Wu W, Jones C, Perwitasari O, Mahalingam S, Tripp RA. MicroRNA Regulation of Human Genes Essential for Influenza A (H7N9) Replication. PLoS One. 2016;11(5):e0155104 PubMed Central PMCID: PMCPMC4864377. doi: 10.1371/journal.pone.0155104 27166678 10.1371/journal.pone.0155104 PMC4864377

[46] Ingle H, Kumar S, Raut AA, Mishra A, Kulkarni DD, Kameyama T, et al The microRNA miR-485 targets host and influenza virus transcripts to regulate antiviral immunity and restrict viral replication. Sci Signal. 2015;8(406):ra126 doi: 10.1126/scisignal.aab3183 26645583 10.1126/scisignal.aab3183

[47] Zhao L, Zhu J, Zhou H, Zhao Z, Zou Z, Liu X, et al Identification of cellular microRNA-136 as a dual regulator of RIG-I-mediated innate immunity that antagonizes H5N1 IAV replication in A549 cells. Sci Rep. 2015;5:14991 PubMed Central PMCID: PMCPMC4598873. doi: 10.1038/srep14991 26450567 10.1038/srep14991 PMC4598873

[48] Rosenberger CM, Podyminogin RL, Navarro G, Zhao GW, Askovich PS, Weiss MJ, et al miR-451 regulates dendritic cell cytokine responses to influenza infection. J Immunol. 2012;189(12):5965–75. PubMed Central PMCID: PMCPMC3528339. doi: 10.4049/jimmunol.1201437 23169590 10.4049/jimmunol.1201437 PMC3528339

[49] Lam WY, Yeung AC, Ngai KL, Li MS, To KF, Tsui SK, et al Effect of avian influenza A H5N1 infection on the expression of microRNA-141 in human respiratory epithelial cells. BMC Microbiol. 2013;13:104 PubMed Central PMCID: PMCPMC3663648. doi: 10.1186/1471-2180-13-104 23663545 10.1186/1471-2180-13-104 PMC3663648

[50] Buggele WA, Krause KE, Horvath CM. Small RNA profiling of influenza A virus-infected cells identifies miR-449b as a regulator of histone deacetylase 1 and interferon beta. PLoS One. 2013;8(9):e76560 PubMed Central PMCID: PMCPMC3784411. doi: 10.1371/journal.pone.0076560 24086750 10.1371/journal.pone.0076560 PMC3784411

[51] Boldin MP, Taganov KD, Rao DS, Yang L, Zhao JL, Kalwani M, et al miR-146a is a significant brake on autoimmunity, myeloproliferation, and cancer in mice. J Exp Med. 2011;208(6):1189–201. Epub 2011/05/11. doi: 10.1084/jem.20101823 21555486 10.1084/jem.20101823 PMC3173243

[52] Bogerd HP, Skalsky RL, Kennedy EM, Furuse Y, Whisnant AW, Flores O, et al Replication of many human viruses is refractory to inhibition by endogenous cellular microRNAs. Journal of virology. 2014;88(14):8065–76. PubMed Central PMCID: PMCPMC4097787. doi: 10.1128/JVI.00985-14 24807715 10.1128/JVI.00985-14 PMC4097787

[53] Aguado LC, Schmid S, Sachs D, Shim JV, Lim JK, tenOever BR. microRNA Function Is Limited to Cytokine Control in the Acute Response to Virus Infection. Cell Host Microbe. 2015;18(6):714–22. PubMed Central PMCID: PMCPMC4683400. doi: 10.1016/j.chom.2015.11.003 26651947 10.1016/j.chom.2015.11.003 PMC4683400

[54] Liu J, Drescher KM, Chen XM. MicroRNAs and Epithelial Immunity. Int Rev Immunol. 2009;28(3–4):139–54. Epub 2009/10/09. doi: 10.1080/08830180902943058 19811319 10.1080/08830180902943058 PMC2764336

[55] Seo GJ, Kincaid RP, Phanaksri T, Burke JM, Pare JM, Cox JE, et al Reciprocal inhibition between intracellular antiviral signaling and the RNAi machinery in mammalian cells. Cell Host Microbe. 2013;14(4):435–45. PubMed Central PMCID: PMCPMC3837626. doi: 10.1016/j.chom.2013.09.002 24075860 10.1016/j.chom.2013.09.002 PMC3837626

[56] Mildner A, Chapnik E, Manor O, Yona S, Kim KW, Aychek T, et al Mononuclear phagocyte miRNome analysis identifies miR-142 as critical regulator of murine dendritic cell homeostasis. Blood. 2013;121(6):1016–27. doi: 10.1182/blood-2012-07-445999 23212522 10.1182/blood-2012-07-445999

[57] Chassin C, Kocur M, Pott J, Duerr CU, Gutle D, Lotz M, et al miR-146a mediates protective innate immune tolerance in the neonate intestine. Cell Host Microbe. 2010;8(4):358–68. Epub 2010/10/19. doi: 10.1016/j.chom.2010.09.005 20951969 10.1016/j.chom.2010.09.005

