# Supplementary figures and images for: miR-144 attenuates the host response to influenza virus by targeting the TRAF6-IRF7 signaling axis
Source: PLoS Pathog. 2017 Apr 5;13(4):e1006305. doi: 10.1371/journal.ppat.1006305 (PMC5393898; doi:10.1371/journal.ppat.1006305)

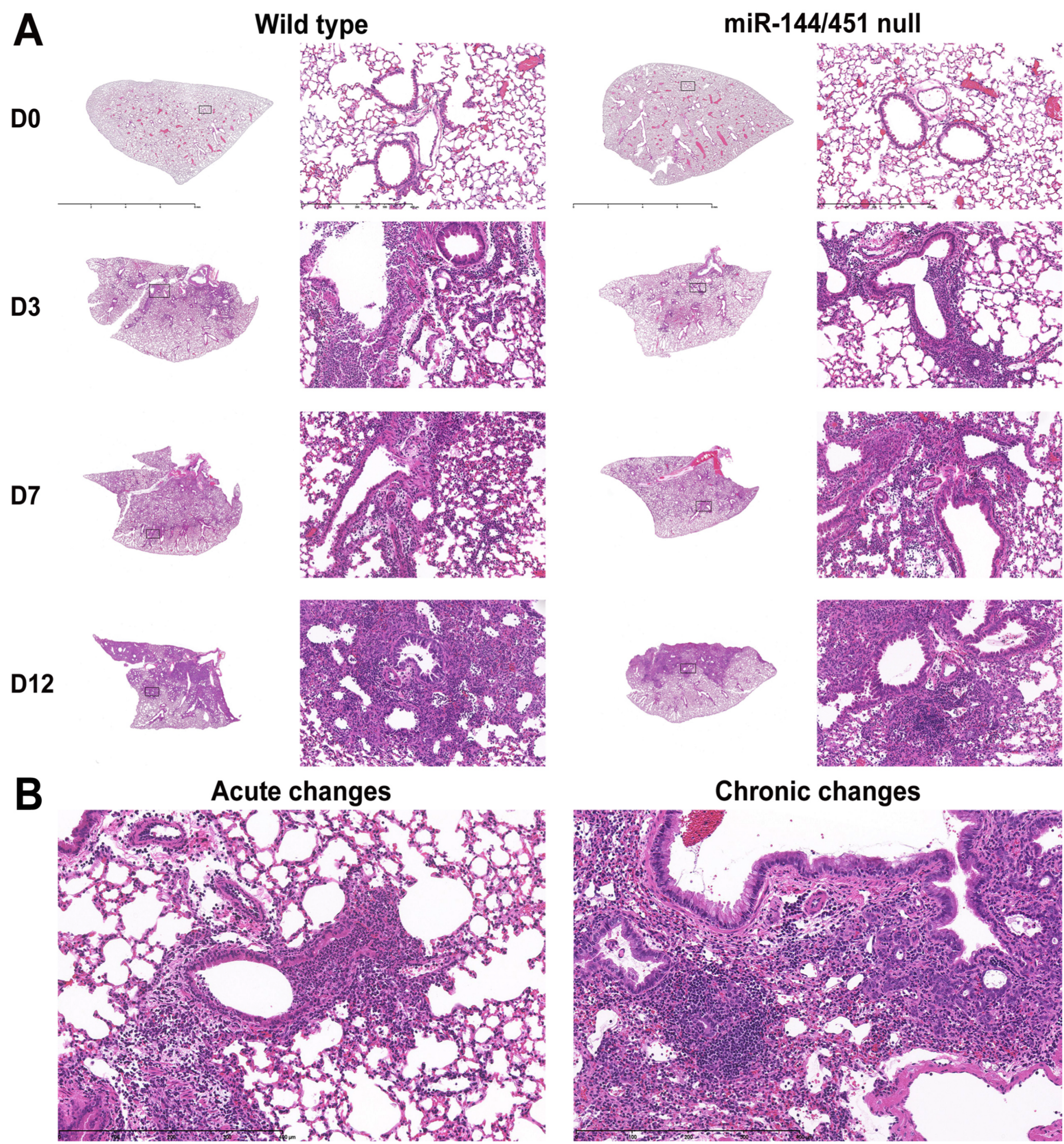

Supplement: S1 Fig — Histology of miR-144/451-/- lungs infected with influenza virus. (A) Representative hematoxylin and eosin-stained lung sections from wild type and miR-144/451-/- mice as indicated. Infection with PR8 (700 pfu) as indicated with D0 representing uninfected controls for comparison of normal morphology in both genotypes. Low power overviews (upper row scale bars = 8mm for all) demonstrate regional distribution of lesions (darker consolidated areas) with decreased extent in the miR-144/451-/- sections. Higher magnifications (upper row scale bars = 400μm for all) correspond to boxed regions within low power overviews. Influenza virus-induced lesions are similar in character but are decreased in severity or extent in miR-144/451-/- with both genotypes demonstrating acute and chronic changes. (B) Representative example of scored acute and chronic changes graphed in Fig 1D (scale bar = 400μm). Acute changes scored include necrosuppurative bronchiolitis (here with regional interstitial spread) and perivascular neutrophils. Other acute lesions in this example from a WT mouse at d3 include intrabronchial necrotic debris, perivascular edema, minimal hemorrhage, and vascular lesions (marginating inflammatory cells, reactive endothelia). Chronic lesions scored included bronchiolar and alveolar hyperplasia, perivascular mononuclear cells and lymphoid aggregates. Other chronic lesions noted in this example from a miR-144/451-/- d12 mouse include mild goblet cell hyperplasia in the large airway and diffuse lymphocytic interstitial pneumonia and alveolitis with mild hemorrhage. (TIF) [file ppat.1006305.s003.tif]

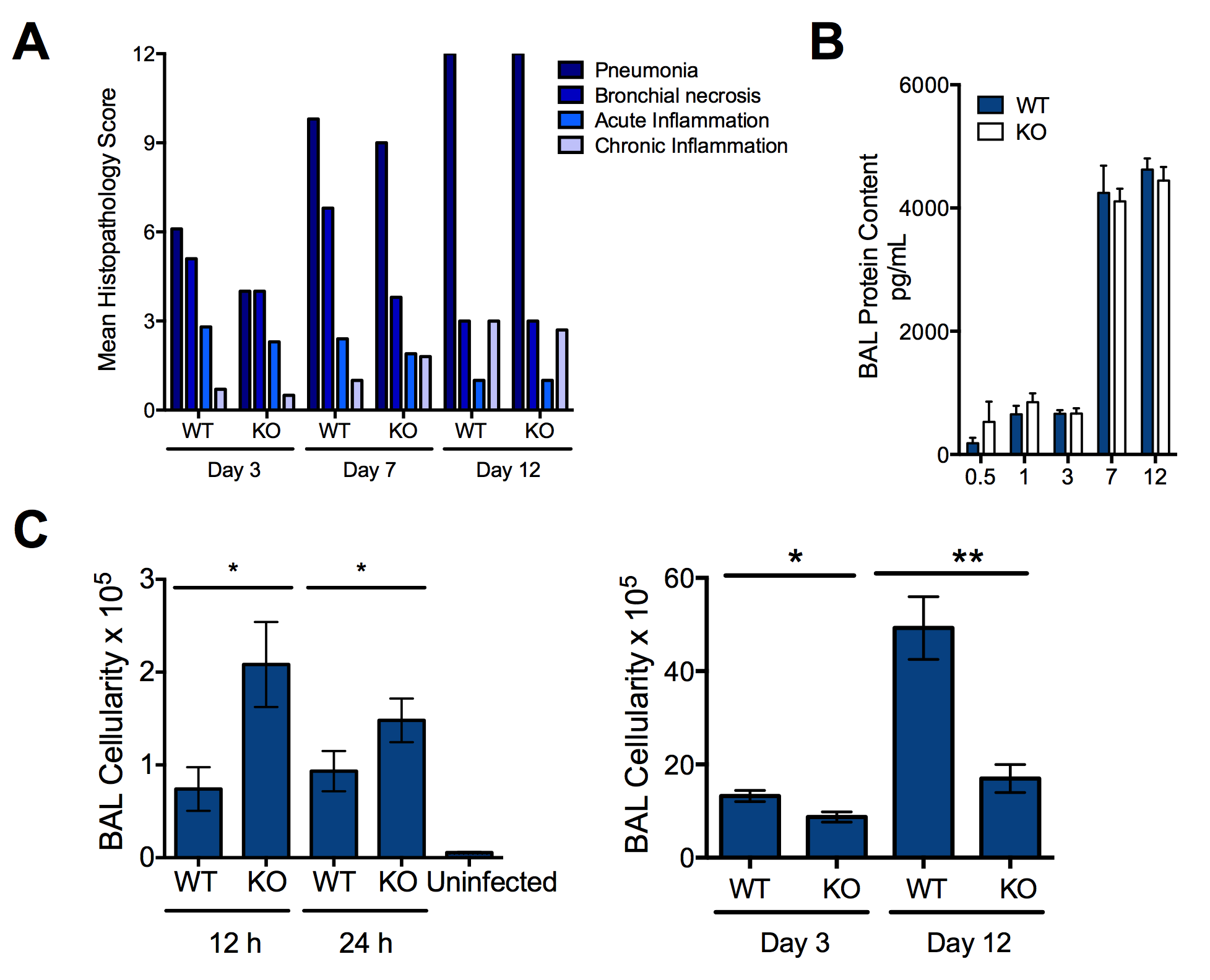

Supplement: S2 Fig — (A) Mice were infected with influenza virus for 3 or 12 d as described in Fig 1. Mean histological scores are shown (scales of 1–4 each for severity and extent of section affected in the most severe manner). Pneumonia = interstitial pneumonia/alveolitis x extent of the most severe changes; Bronchial necrosis = necrotizing bronchiolitis x extent of the most severe changes; Acute inflammation = necrosuppurative bronchiolitis + perivascular neutrophils; Chronic inflammation = bronchial hyperplasia + alveolar hyperplasia + perivascular mononuclear cells + lymphoid aggregates. 3 d: n = 13–14, 7 d, n = 4–6, 12 d: n = 4–6. (B) Protein content as a surrogate metric of lung barrier function was quantified by BCA assay of BAL fluid obtained from 4–14 mice per time point; p>0.1 for all time points. (C) The number of inflammatory cells obtained in the BAL was enumerated and means ±SEM (n = 4–6) are representative of 1–3 independent experiments. (TIFF) [file ppat.1006305.s004.tiff]

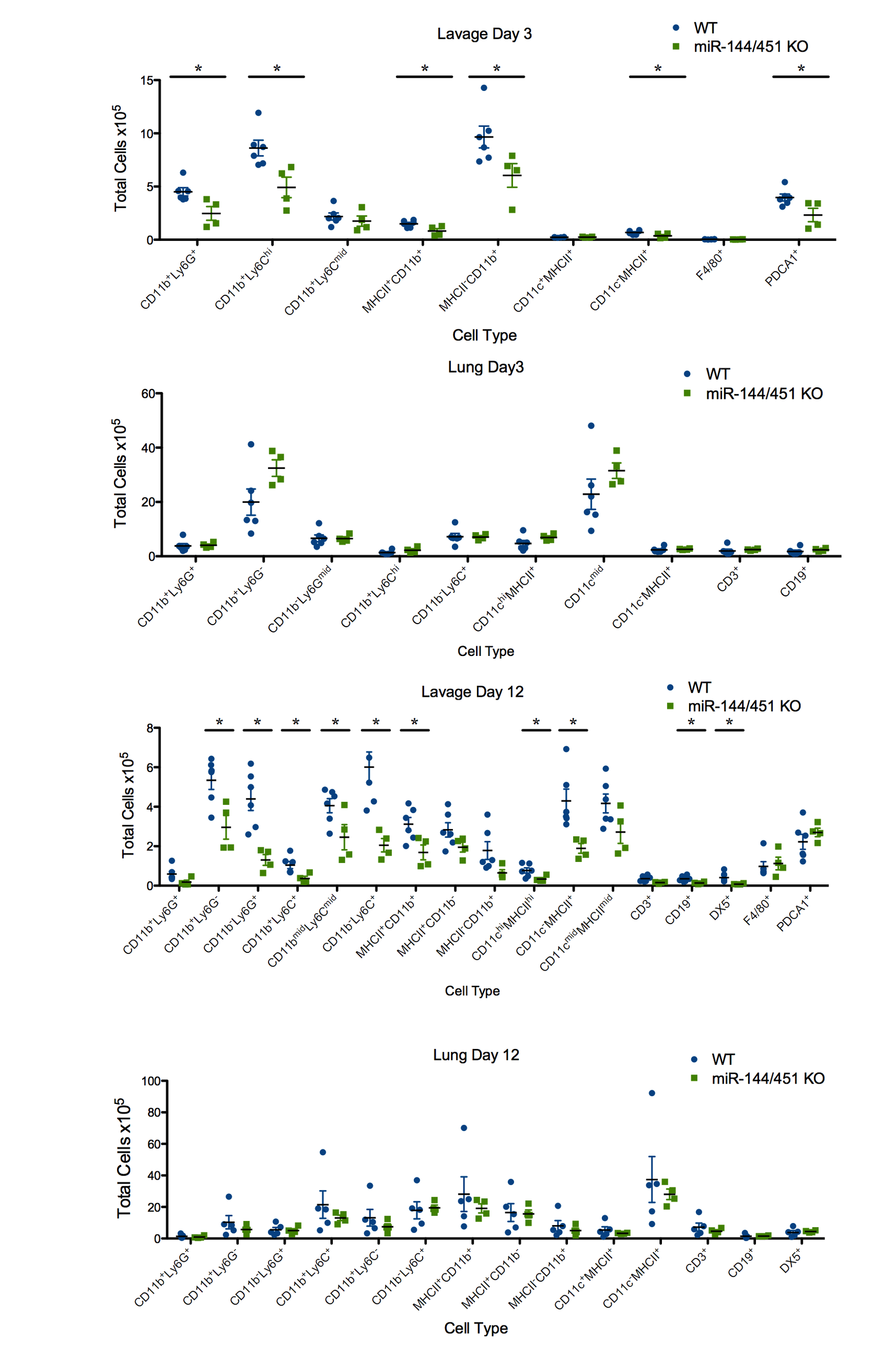

Supplement: S3 Fig — Cells collected by bronchoalveolar lavage or enzymatic dissociation of infected lung tissue were stained with a panel of cell lineage-specific antibodies and analyzed by flow cytometry. Medians are plotted; * p<0.05. (TIF) [file ppat.1006305.s005.tif]

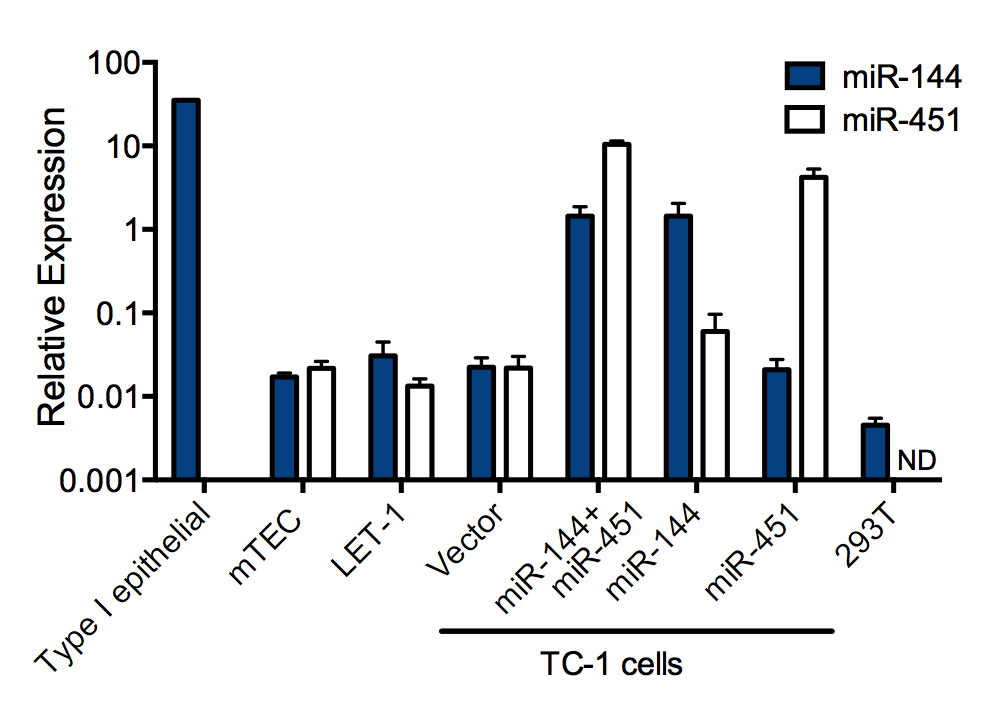

Supplement: S4 Fig — Expression of miR-144 and miR-451 in primary type I lung epithelial cells was compared to the expression level in primary polarized tracheal epithelial cells (mTEC), cultured primary lung alveolar epithelial type I cells (LET1), mouse TC-1 epithelial cell lines with or without stable transduction of microRNAs, and 293T cells. Expression was measured by qRT-PCR and plotted relative to sno-202 expression. Means ±SEM are shown for 3–8 cellular samples. ND = not determined. (TIFF) [file ppat.1006305.s006.tiff]

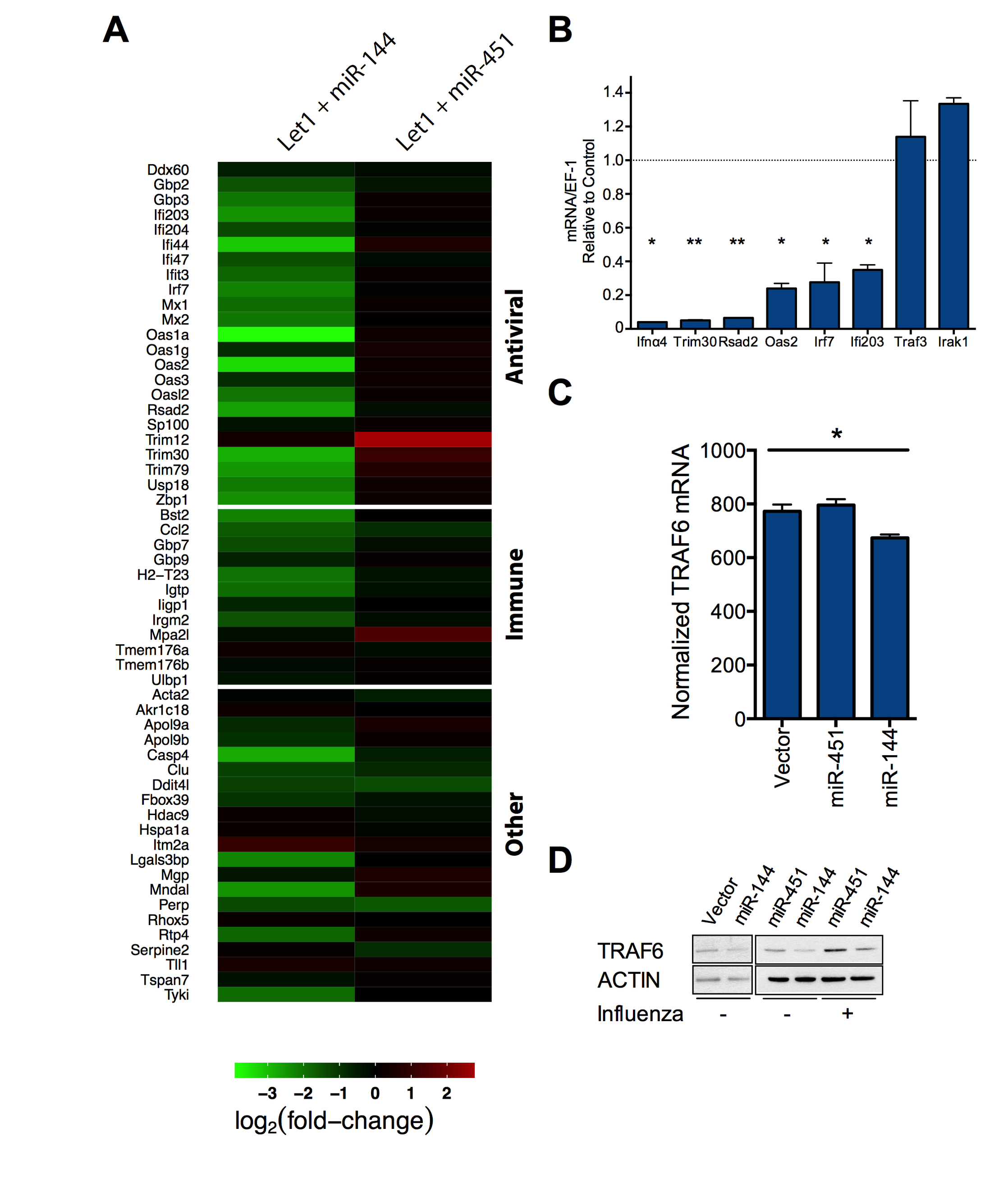

Supplement: S5 Fig — (A) Microarray transcriptional analysis of LET1 cells stably expressing miR-144, miR-451, or vector alone were infected with influenza virus for 1 h. The heatmap depicts fold-change relative to a vector control for the set of genes whose expression in TC-1 cells following influenza virus infection was affected more than 2-fold (p<0.05) by miR-144/451 over-expression (Fig 3A) with red and green representing up- and down-regulation respectively. Mean relative intensities for 5 (miR-144) or 2 (miR-451) independent experiments using 5 (vector alone) control samples are shown. Complete expression data is available at GEO (GSE50742). (B) qRT-PCR was performed as described in Fig 3B on influenza-infected LET1 cells, with gene expression in cells stably expressing miR-144 alone shown relative to cells expressing vector alone; n = 2 and representative of 3 experiments. (C) TRAF6 expression in the cells described in A was measured by Agilent microarray. Means ± SEM are plotted for n = 5 (miR-144 and vector) or n = 2 (miR-451); *p = 0.013. (D) Reduced TRAF6 protein in TC-1 cells expressing miR-144 compared to those expressing miR-451 or vector alone pre- or 6 h post-infection with influenza virus. TRAF6 protein expression relative to actin was quantified by densitometry of Western blots: Uninfected: miR-451, 0.70; miR-144, 0.37; Infected: miR-451: 1.0; miR-144: 0.66. Representative Western blot for the 3 experiments graphically displayed in Fig 4G. (TIF) [file ppat.1006305.s007.tif]

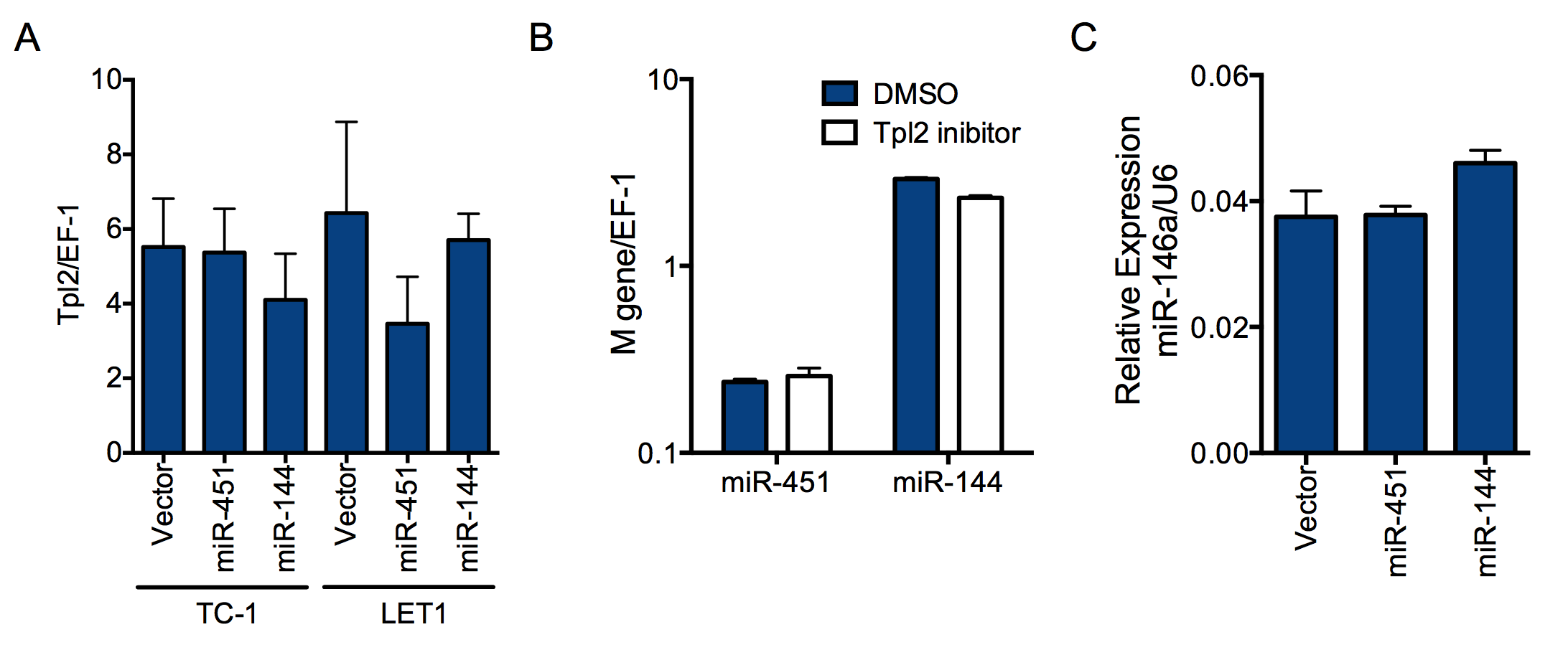

Supplement: S6 Fig — (A) Ectopic expression of miR-144 in TC-1 or LET1 cells did not alter Tpl2/Map3K8 expression. qRT-PCR gene expression normalized by EF-1 levels and plotted in arbitrary units; means ±SEM (n = 2–10). (B) Chemical inhibition of the Tpl2 kinase did not increase influenza virus replication over 24 h in LET1 cells overexpressing miR-144 or miR-451 (control), as assessed by qRT-PCR of M gene normalize by EF-1; means ±SEM (n = 3–4). (C) Expression of miR-146a is equivalent in LET1 cells expressing miR-144 compared with cells expressing miR-451 as a control, or vector alone. miR-146a measured by qRT-PCR is plotted in arbitrary units relative to U6 expression. Means ± SEM for 2–4 samples are shown. (TIFF) [file ppat.1006305.s008.tiff]
